# Comparing the Diagnostic Performance of ECG Gated versus Non-Gated CT Angiography in Ascending Aortic Dissection: A GRRAS Study

**DOI:** 10.3390/tomography8050201

**Published:** 2022-09-28

**Authors:** Razvan G. Budeanu, Christian Broemmer, Anamaria R. Budeanu, Marian Pop

**Affiliations:** 1 Emergency County Hospital Târgu Mureș, 540136 Targu Mures, Romania; 2 ME1 Department, “George Emil Palade” University of Medicine, Pharmacy, Science and Technology of Targu Mures, 540142 Targu Mures, Romania; 3 Radiology and Medical Imaging Department, Emergency Institute for Cardiovascular Disease and Heart Transplant of Targu Mures, 540136 Targu Mures, Romania

**Keywords:** inter-rater reliability, radiology, ECG-gated, CTA, ascending aortic dissection

## Abstract

Rationale and Objective: Thoracic CT angiography (CTA) for ascending aortic dissection, a life-threatening emergency, is performed routinely without Electrocardiographic (ECG) gating, therefore allowing the apparition of a pulsation artefact. We aimed to evaluate and compare the diagnostic performance, the inter and intra-reporter agreement of ECG gated CTA and non-ECG gated CTA for detecting ascending aortic dissection, considering their training level. Our hypothesis is that ECG gated CTA has superior diagnostic accuracy for ascending aortic dissection compared to non-gated CTA. Materials and Methods: We collected data using 24 questions survey using clinically validated CT examinations. Sixty-six respondents (medical students, radiology residents, and consultants) blinded to the actual diagnosis independently evaluated the images pertaining to the presence of ascending aortic dissection. The reference standard was represented by clinical and imaging diagnosis. Inter-rater and inter-group concordance was evaluated; the agreement with reference tests was calculated and assessed as a function of reporters’ training level. Results: Reporters’ ascending aortic dissection assessment showed a better correlation with the reference standard in the ECG gated CTA. The inter-rater correlation was higher in the ECG gated CTA compared to non-ECG gated CTA. Observers’ confidence for diagnosing ascending aortic dissection was higher in the ECG gated CTA. Statistically significant differences (*p* < 0.05) were found between different training levels when assessing non-ECG gated examinations. Conclusions: ECG gated CTA shows a higher diagnostic performance for ascending aortic dissection than non-ECG gated CTA, regardless of the reporters’ training level.

## 1. Introduction

Computed tomography (CT) is the gold standard for evaluating acute thoracic aortic diseases [1], with CT angiography (CTA) replacing diagnostic angiography [2]. CTA is widely available, is near the emergency room, has high sensitivity and specificity for detecting acute thoracic aortic diseases, and can detect other findings, enabling it to rule out a plethora of diseases [2,3]. 

Despite being widely used to diagnose ascending aortic dissection [4,5,6,7,8,9], CTA may provide false-positive results, as factors such as streak artifacts, pericardial recesses, the left brachiocephalic vein, thickened pleura, and, most importantly, motion artifacts from the pulsating aorta may simulate an intimal flap or false channel [10,11]. Electrocardiographic (ECG) gating/triggering eliminates cardiac pulsation motion artifacts, thus improving the diagnosis of an acute aortic dissection, enabling clinicians to precisely localize and characterize the site of the primary intimal tear, with important clinical implications [12,13].

Although it is well known that ECG gated CTA produces better image quality with fewer motion artifacts than non-ECG gated CTA [14,15], it is rarely used in routine chest CTA. 

Previous studies have comparing ECG gated vs. non-ECG gated CTA examinations found that, even when using newer machines, with high pitch, the quality of ascending aortic CTA is vastly improved by using gating [16,17,18,19].

Moreover, due to increased image quality, removal of pulsation artifacts and measurement reproducibility the ECG gated CTA represents the current standard for pre-TAVI procedures but not yet for acute aortic syndromes [20,21].

This study aimed to evaluate and compare the diagnostic performance, the inter and intra-reporter agreement and reporters’ confidence of ECG gated CTA and non-ECG gated CTA for detecting ascending aortic dissection, considering their training level. Our hypothesis is that ECG gated CTA has superior diagnostic accuracy for ascending aortic dissection compared to non-gated CTA.

## 2. Materials and Methods

This study was approved by the Ethics Committee of the Emergency Institute for Cardiovascular Diseases and Heart Transplant of Târgu Mureș 9656/2021. General consent was obtained from all patients at the time of their CT scan performed to obtain the images used in the survey. Implied consent was obtained by respondents on the submission of the anonymous survey. All steps were performed in agreement with the applicable guidelines and regulations at our medical center.

### 2.1. Participants

A 24-question electronic survey designed using Google Forms was distributed via professional networks from October 2021 to January 2022 to board-certified radiologists, radiology trainees, and medical students. The segmentation of target groups has been performed based on their training, medical students undergoing 1 semester of radiology lectures, the radiology trainees (residents) who were invited having more than 1 year of radiology practice, and the specialist group representing board-certified individuals (5+ years of radiology training and practice). 

### 2.2. Index Tests

CTA images were obtained using 64 slice CT machines, with BSA-adjusted kV and automatic mA. The contrast agent was administered using power-injector, with a flowrate of 4 to 5 mL/s, depending on the venous access gauge. The patients were in the supine position, with inspiratory breath-hold command. The bolus-tracking technique was used to start the examination. Axial images were reconstructed using a slice thickness of 0.6/0.625 mm.

**Figure 1:**
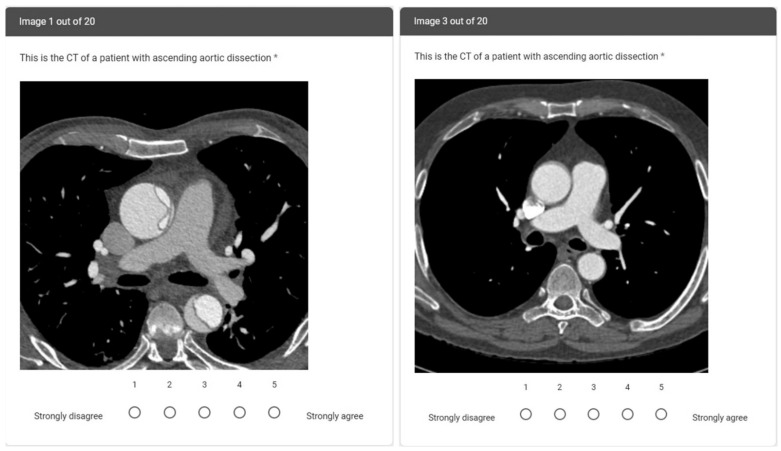
Sample survey questions from patients with ECG gating and aortic dissection (**left**) and without ECG gating and without aortic dissection (**right**). On the ECG gated CTA image, the ascending aortic dissection flap is clearly visible. On the non-ECG gated CTA image, there is movement around the ascending aorta, leading to challenges in diagnostic confidence. * was used to mark the question as mandatory.

The images were anonymized and randomized. We used the images of fourteen patients: seven with confirmed and seven with excluded ascending aortic dissection diagnosis. In each subgroup, we included examinations performed with and without ECG gating. Six images were used twice, as separate questions, to assess intra-rater reliability, thereby increasing the total number of image-related questions to twenty. Clinical information was not available to the participants. The readers independently interpreted the images and evaluated the certainty regarding the presence or absence of ascending aortic dissection (Likert scale from 1 = Strongly disagree to 5 = Strongly agree). See Figure 1 for sample questions. Respondent characteristic data were also recorded: age, gender, level of training, and years of CT experience.

### 2.3. Reference Tests 

All the images were carefully selected following consensus reading by 2 radiologists (board-certified with 10+ years of experience in cardiovascular imaging and trainee with 1+ years of experience). The complete electronic health records, including the clinical course of the patient, were available to the radiologists. The clinical data and patients’ history up to discharge were used to double-check the radiology readings.

### 2.4. Statistical Analysis

Data were analyzed using Microsoft Office Excel (Microsoft Corporation. (2018). Microsoft Excel. Retrieved from https://office.microsoft.com/excel) and IBM SPSS Statistics 26 (IBM SPSS Statistics for Windows, version 26, IBM Corp., Armonk, NY, USA). In compliance with the Guidelines for Reporting Reliability and Agreement Studies (GRRAS) criteria [22], the variability of diagnosing an aortic dissection was analyzed by determining the inter-rater and intra-rater agreement. Inter-rater reliability is the degree of agreement between 2 or more evaluators, indicating the coherence of the implementation of a scoring system. Inter-rater reliability was calculated using the interclass correlation coefficient (ICC), which is a method for determining agreement and is expressed as a relative measure of the explained variance of the total random variance [23]. The two-way mixed effects, absolute agreement, and single measure approach were chosen. The ICC values were interpreted as follows: >0.80 was considered very good, 0.61–0.80 was considered good, 0.41–0.60 was considered moderate, 0.21–0.40 was considered fair, and 0.21 was considered poor [24]. Continuous variables with a normal distribution are presented as mean ± standard deviation. Median and interquartile ranges were used as measures of central tendency for continuous variables with non-normal distribution. A Mann–Whitney test was used for comparison of data with non-normal distribution. Categorical data were expressed as percentage frequencies. A p-value < 0.05 was considered to denote statistical significance.

## 3. Results 

Sixty-six respondents submitted the survey (M: F = 0.78), with an average respondent age of 28 years (22 to 43 years). Most of the respondents were students (59.1%), followed by residents (22.72%) and consultants (18.18%). Only five participants had more than five years of CT experience (7.58%), while more than half had no prior CT training (54.54%). Full demographic details are in Table 1.

### 3.1. Agreement with Reference Tests 

**Figure 2:**
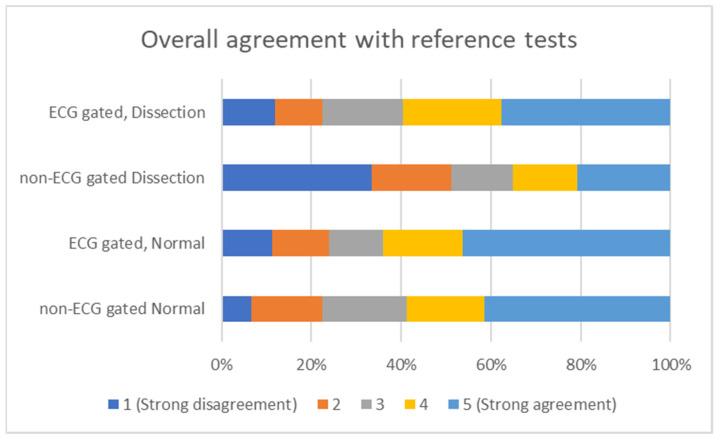
Overall agreement with reference tests, depending on the usage of ECG gating for CTA image acquisition.

Overall agreement with the reference tests varied depending on the presence of gating, with the strongest agreement for ECG gated CTA of normal images (46.36%) and the worst for non-ECG gated CTA of normal images (6.67%). See Figure 2.

**Figure 3:**
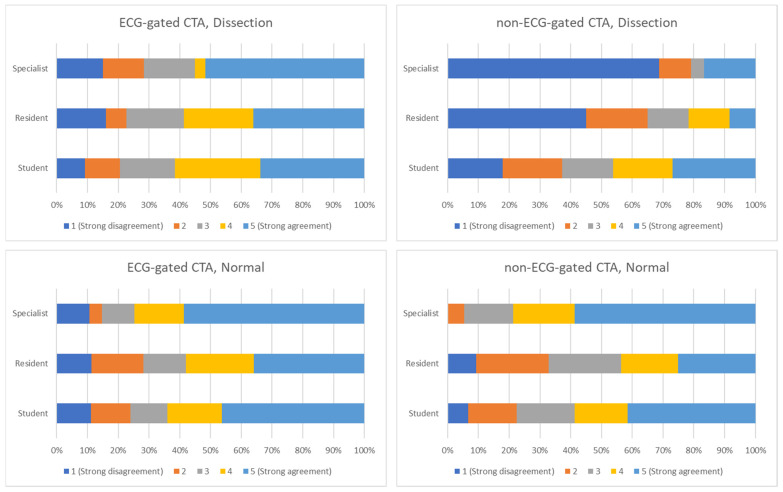
Agreement between different observers and type of image as answering the question “This is the CT of a patient with ascending aortic dissection”. (**Upper-left panel**): CTA performed using ECG gating, with positive dissection diagnosis. (**Upper-right panel**): CTA performed using without ECG gating, positive dissection diagnosis. (**Lower-left panel**): CTA performed using ECG gating, normal study. (**Lower-right panel**): CTA performed without ECG gating, normal study.

Absolute concordance with the reference tests diagnosis was highest for specialists working on ECG gated CTA images and the lowest for specialists working with non-ECG gated CTA images (68.75%)- see Figure 3.

Quantitative comparison between the three categories of responders showed statistically significant differences between study participants in non-ECG gated images (*p*-value between 0.001 and 0.0365) and between students’ responses when compared with residents (*p* = 0.0003) and consultants (*p* = 0.0013).

### 3.2. Inter-Rater Reliability 

The inter-rater reliability for each of the three classes of evaluators (students, residents, and specialists) is shown in Table 2. The highest ICC was in the specialist group for ECG gated images (0.498), and the lowest was in the students’ group for the non-ECG gated images (0.173). Results ranged from moderate to poor. The lowest ICC value for ECG gated images (0.319—students group) was higher than the highest ICC value for the non-ECG gated images group (0.227—specialists group).

When comparing different training levels, the ICCs ranged from good—0.657 (residents vs. specialists) to poor 0.1 (students vs. specialists, non-ECG gated images). The full data on the comparison can be found in Table 3.

### 3.3. Intra-Rater Reliability 

The ICCs for intra-rater reliability ranged from very good 0.82 in the case of specialists reporting ECG gated images to good—0.593 for specialists reporting non-ECG gated images (Table 4). 

## 4. Discussion

We demonstrated that, when comparing ECG gated CTA vs non-ECG gated CTA, the diagnostic performance, the inter and intra-reporter agreement, and reporters’ confidence for detecting ascending aortic dissection were increased, regardless of their training level. 

As expected, years of experience were an important predictor of correct diagnosis. Our results concur with previous research [25,26]; specialists had higher numbers of correct answers and higher ICC values in both intra- and inter-rater reliability compared to students and residents, although the residents’ results were very close to the specialists’ results. 

A high value of ICC indicates a lower variance in responders answers, meaning they were more likely to agree with each other (when interpreting inter-reliability) or with themself (when interpreting intra-reliability). A low value indicates that the respondents were unsure of their answers, changing their answers when the questions are repeated (intra-reliability) or giving different answers than the other responders (inter-reliability).

When comparing the ICCs for ECG and non-ECG gated images, we found higher ICC scores in all groups, which might indicate that ECG gated thoracic CT is easier to interpret to reach an accurate diagnosis; this corresponds to changes made to practice and national recommendations [27] regarding the use of ECG gating in CTA.

Moreover, when comparing ECG gated to non-ECG gated thoracic CT, the ICC was more than double in all study groups (Table 3 and Table 4), demonstrating that ECG gated images are more reliable for interpreting thoracic CT. Furthermore, the rate of incorrect answers was lower for ECG gated images compared to non-ECG gated images for both single and repeated images. 

The intra-rater reliability was very good, indicating that the participants were sure about their answers, though the students were the most unsure and tended to modify their responses.

Regarding quantitative comparison, statistically significant differences between respondents in non-ECG gated images and the lack of this difference in ECG gated images might imply that ECG gated CT provides a more accurate perspective of the pathology, with an increased chance of a correct diagnosis. The statistical difference between student-residents (*p* = 0.0003) and student-specialists (*p* = 0.0013) in non-aortic dissection ECG gated images, combined with a lack of statistical difference between resident-specialists (*p* = 0.7942) indicate a higher rate of false-positive results in groups without sufficient experience in interpreting the images.

Different contrast agents may provide various levels of opacification [28], and, using a protocol targeted on the estimated body mass index [29], in our study, the images were acquired using the same contrast agent and the same protocol; therefore, they should provide similar enhancement and no differences in terms of dissection flap identification.

Minimizing radiation exposure is a critical issue in patients with acute aortic syndromes (AAS), as these patients are often scheduled for follow-up examinations and even interventional treatment in selected cases. 

Retrospectively ECG gated multidetector computed tomography (MDCT) is usually performed with low pitch values for high data oversampling, which enables image reconstructions at any point during the cardiac cycle; however, this comes with a major disadvantage of increased radiation dose due to the acquisition of highly overlapping slices. In contrast, non-gated MDCT employs high pitch values and therefore exposes the patient to a substantially lower radiation dose, making this technique more susceptible to motion artifacts [30]. Dose-related concerns might be solved by prospective ECG triggering, though this method also carries some costs [31].

### Limitations

A limitation of our study is the potential for bias in recruiting responders for the convenience samples used. Other limitations, common to most online surveys, are related to low participation rates among occasional Internet users, making it difficult to gather large numbers of respondents, which may affect the generalization of the results. Furthermore, the data presented in this study are self-reported and partially dependent on the honesty of the participants and the hardware used for image evaluation. However, the findings do provide valuable information regarding the value of using ECG-guided CTA.

## 5. Conclusions

ECG gated CTA plays a significant role in the correct diagnosis of ascending aortic dissection with the benefit of removing the motion artifact of the ascending aorta and increasing diagnostic precision. 

ECG gated CTA shows a higher diagnostic performance for ascending aortic dissection than non-ECG gated CTA regardless of the reporters’ training level.

Statistically significant differences were found between the different target groups when reporting non-ECG gated images.

## Figures and Tables

**Table 1:** Demographic details of respondents. IQR—Interquartile range.

	Medical Students (*n* = 39)	Radiology Residents (*n* = 15)	Radiology Consultants (*n* = 12)
Median age (IQR)	25 (24–26)	28 (27–29.5)	37 (34.5–38)
Male to female ratio	1.16	0.36	0.5
Years of CT training: Less than 1 1 to 5 years More than 5 years	30 9	6 9	7 5

**Table 2:** Inter-rater reliability for the three groups of reporters, detailed by the usage of gating for image acquisition.

	ECG Gated Images ICC (95% CI)	Non-ECG Gated Images ICC (95% CI)
Students	0.319 (0.162–0.644)	0.173 (0.078–0.417)
Residents	0.481 (0.269–0.783)	0.26 (0.117–0.552)
Specialists	0.498 (0.273–0.797)	0.227 (0.087–0.52)

ICC intraclass correlation coefficient, CI confidence interval.

**Table 3:** Inter-rater reliability when comparing reporters with different training levels (students, residents, and specialists).

	ECG Gated Images ICC (95% CI)	NON-ECG Gated Images ICC (95% CI)
Students vs. Residents	0.425 (0.272–0.556)	0.166 (0.006–0.317)
Students vs. Specialists	0.452 (0.275–0.596)	0.1 (0.01–0.117)
Residents vs. Specialists	0.657 (0.535–0.752)	0.291 (0.128–0.439)

ICC intraclass correlation coefficient, CI confidence interval.

**Table 4:** Intra-rater reliability ICC for the three groups of reporters, detailed by the usage of gating for image acquisition (two-way mixed, absolute agreement, single measures).

	ICC ECG Gated Images (95% CI)	ICC Non-ECG Gated Images (95% CI)
Students	0.692 (0.584–0.775)	0.637 (0.516–0.733)
Residents	0.711 (0.53–0.83)	0.807 (0.675–0.889)
Specialists	0.82 (0.676–0.9)	0.593 (0.332–0.499)

ICC intraclass correlation coefficient, CI confidence interval.

## Data Availability

Not applicable.
